# *Ophiocordyceps formosana* improves hyperglycemia and depression-like behavior in an STZ-induced diabetic mouse model

**DOI:** 10.1186/s12906-016-1278-7

**Published:** 2016-08-24

**Authors:** Chao-Wei Huang, Tzu-Wen Hong, Ying-Jing Wang, Ko-Chien Chen, Ju-Chun Pei, Tai-Yuan Chuang, Wen-Sung Lai, Sheng-Hong Tsai, Richard Chu, Wei-Cheng Chen, Lee-Yan Sheen, Satoru Takahashi, Shih-Torng Ding, Tang-Long Shen

**Affiliations:** 1 Department of Animal Science and Technology, National Taiwan University, Taipei, 10617 Taiwan; 2 Mucho Biotechnology Inc., Taipei, 10684 Taiwan; 3 Department of Plant Pathology and Microbiology, National Taiwan University, Taipei, 10617 Taiwan; 4 Department of Psychology, National Taiwan University, Taipei, 10617 Taiwan; 5 Department of Athletics, National Taiwan University, Taipei, 10617 Taiwan; 6 Institute of Biotechnology, National Taiwan University, Taipei, 10617 Taiwan; 7 Institute of Food Science and Technology, National Taiwan University, Taipei, 10617 Taiwan; 8 Graduate School of Comprehensive Human Sciences, University of Tsukuba 1-1-1, Tennodai, Tsukuba, 305-8575 Japan

**Keywords:** *Ophiocordyceps formosana*, Insulin insensitivity, Depression

## Abstract

**Background:**

A newly defined Cordyceps species, *Ophiocordyceps formosana* (*O. formosana*) has been implicated in multitudinous bioactivities, including lowering glucose and cholesterol levels and modulating the immune system. However, few literatures demonstrate sufficient evidence to support these proposed functions. Although the use of *Cordyceps spp.* has been previously addressed to improve insulin insensitivity and improve the detrimental symptoms of depression; its mechanistic nature remains unsettled. Herein, we reveal the effects of *O. formosana* in ameliorating hyperglycemia accompanied with depression.

**Methods:**

Diabetes was induced in mice by employing streptozotocin(STZ), a chemical that is toxic to insulin-producing β cells of the pancreas. These streptozotocin (STZ)-induced diabetic mice showed combined symptoms of hyperglycemia and depressive behaviors. Twenty-four STZ-induced mice were randomly divided into 3 groups subjected to oral gavage with 100 μL solution of either PBS or 25 mg/mL *Ophiocordyceps formosana* extract (OFE) or 2 mg/mL rosiglitazone (Rosi, positive control group). Treatments were administered once per day for 28 days. An additional 6 mice without STZ induction were treated with PBS to serve as the control group. Insulin sensitivity was measured by a glucose tolerance test and levels of adiponectin in plasma and adipose tissue were also quantified. Behavioral tests were conducted and levels of monoamines in various brain regions relating to depression were evaluated.

**Results:**

HPLC analysis uncovered three major constituents, adenosine, D-mannitol and cordycepin, within *O. formosana* similar to other prestigious medicinal *Cordyceps spp..* STZ-induced diabetic mice demonstrated decreased body weight and subcutaneous adipose tissue, while these symptoms were recovered in mice receiving OFE treatment. Moreover, the OFE group displayed improved insulin sensitivity and elevated adiponectin within the plasma and adipose tissue. The anti-depressive effect of OFE was observed in various depression-related behavior tests. Concurrently, neurotransmitters, like 5-HT and dopamine in the frontal cortex, striatum and hippocampus were found to be up-regulated in OFE-treated mice.

**Conclusions:**

Our findings illustrated, for the first time, the medicinal merits of *O. formosana* on Type I diabetes and hyperglycemia-induced depression. OFE were found to promote the expression of adiponectin, which is an adipokine involved in insulin sensitivity and hold anti-depressive effects. In addition, OFE administration also displayed altered levels of neurotransmitters in certain brain regions that may have contributed to its anti-depressive effect. Collectively, this current study provided insights to the potential therapeutic effects of *O. formosana* extracts in regards to hyperglycemia and its depressive complications.

## Background

Type I diabetes (T1D) is an autoimmune disease caused by an immune response against the β cell antigens in the pancreas, which results in a lack of insulin, but an increase in blood and urine glucose levels [1]. In 2014, an estimate of 3 million people was reported to have T1D in the USA [2]. Current therapies for T1D place strong emphasis on insulin replacement or transplantation of insulin secreting tissues; however, numerous limitations and complications still remain. However, Gunawardana et al. [3] argued that adipose tissues are able to maintain glucose homeostasis in the absence of insulin; while adipokines have been shown to ameliorate both type 1 and 2 diabetic phenotypes in the absence of exogenous insulin by potentiating insulin receptor sensitivity [3–5]. Past literatures have shown a positive correlation between adiponectin levels and insulin sensitivity in T1D patients [6]; moreover, higher levels of adiponectin is found to be associated with lower prevalence of metabolic syndrome in T1DM [7]. In addition, STZ-induced type 1 diabetic mice displayed ameliorated hyperglycemic symptoms following adiponectin gene delivery into mice [3]. Consequently, an increase in the level of plasma adiponectin was found to improve insulin resistance [4].

Depression is commonly linked with hyperglycemia in T1D and T2D patients [8, 9] and is found to be more prevalent in T1D patients according to the Beck Depression Inventory Score [10]. Previous studies suggest that approximately 15 % of diabetic patients risk complications of clinical depression [8]. Thus, patients with type I diabetes also require the increased use of antidepressants [11]. Selective serotonin re-uptake inhibitors (SSRIs) are widely used as the first-line treatment for depression; however, several side effects, including nausea, anorexia and sexual dysfunction, are commonly observed [12, 13]. Unfortunately, SSRIs can lead to failure of glycemic control in diabetic patients. On the other hand, tricyclic antidepressants (TCAs) can also contribute to hyperglycemia in humans and mice [14]. Interestingly, a study showed that diabetic patients treated with Rosi for 12 weeks exhibited a significant decline in depressive severity as evaluated by the Hamilton Depression Rating Scale and the Clinical Global Impression Scale [15]. However, the side effects of Rosi include increase risk of myocardial infarction [16]. Thus, an effective alternative treatment with little or no side effects for T1D and depression-related complications is still in demand.

Many psychiatric disorders, such as anxiety and depression, can be ascribed to deficits in monoamine neurotransmitters [17, 18]. Thus, many antidepressants are aimed toward modulating the neurotransmission system by increasing the content and amount of monoamines (e.g., dopamine, 5-hydroxytryptamine, and norepinephrine) in the limbic regions of the brain. The hypothalamic serotonin is increased during insulin-induced hypoglycemia[19], indicating that glycemic homeostasis may be beneficial for ameliorating depression. As a result, adipokines (e.g., leptin and adiponectin) can promote insulin sensitivity as well as alleviate depressive behaviors [20, 21].

Some *Cordyceps spp.* have been used in traditional Chinese medicine (TCM) to lower blood pressure, glucose and cholesterol, as well as to modulate immune function [22]. Aside from ameliorating metabolic dysfunctions, *Cordyceps spp.* also improve psychological disorder like insomnia, dysphoria and sleep [23]. Even though the scientific reports to address *Cordyceps spp*. effectiveness as an antidepressant regimen [24] and an improvement of both hyperglycemia and depression in rodents treated with *Ophiocordyceps sinensis* [25], there is still inadequate number of scientific evidence in support of clinical use.

Our present study reported a close relationship in phylogenetic and secondary metabolism profiles between *O. sinensis* and *O. formosana* prompting the evaluation of the effects of *O. formosana* on hyperglycermia and depression. Herein, we addressed potential medicinal effects of *O. formosana* on hyperglycemia depression. By employing an STZ-induced diabetes mouse model, we identify the effects of OFE in comparison to Rosi via measuring glucose tolerance, and assessing the anxiety-like and depression-like behaviors of mice. The expressions of adiponectin in plasma and adipose tissue in situ were examined. In addition, the levels of several neurotransmitters were measured to correlate with the changes in psychological behaviors in response to *Ophiocordyceps formosana* treatments in STZ-induced diabetic mice.

## Methods

### Cultivation and extraction of Ophiocordyceps formosana

An epitypified *Ophiocordyceps formosana* (OF) was collected, identified, cultivated and maintained by Wang et al., as described in our previous study [26]. The voucher specimen of *Ophiocordyceps formosana* has been deposited in Leibniz-Institut DSMZ-Deutsche Sammlung von Mikroorganismen und Zellkulturen GmbH designated as *Ophiocordyceps formosana* MUCHO 815-DSM 32000. *Ophiocordyceps formosana* (OF) was maintained and cultivated according to our previous report [26]. OF was grown on potato dextrose agar (PDA) containing 0.4 % potato starch, 2 % dextrose, and 1.5 % agar (Difco Becton Dickinson, Sparks, MD, USA) at 25 °C for 28 days. The colonies were then collected, lyophilized, and pulverized by a homogenizer (SH100, KURABO International Co., Tokyo, Japan) at 1300 rpm. The preparation of *Ophiocordyceps formosana* extracts (OFE) was proceeded by the standard operating procedure as previously described [26, 27]. In brief, 1 gram of OF powder was mixed with deionized water at a ratio of 1:40 (w/v). Thereafter, the extractions were carried out in a hot water bath at 50 °C for 2 h along with sonication during the first 30 min. The samples were then centrifuged at 3000 g for 20 min. The supernatant fractions were sterilized through 0.22 μm filters (Millipore, USA), and stored at −80 °C. Each batch of OFE was subjected to content analyses by high-performance liquid chromatography (D2000 system, Hitachi Co., Tokyo, Japan).

### HPLC analyses of Ophiocordyceps formosana extract (OFE)

The quality control of OFE was conducted by HPLC (D2000 system, Hitachi Co., Tokyo, Japan) utilizing a photodiarray detector (Primaide 1430, Hitachi Co., Tokyo, Japan). The analyses were performed using a RP-18 column (150252 Purospher®STAR RP-18 endcapped (5 μm) LiChroCART®250-4, Merck Co., New Jersey, USA.) at a flow rate of 1 mL/min. The mobile phase was 20 % methanol in H_2_O and the absorbance was detected at 260 nm. Two components in *Cordecyps spp*, adenosine (A9252, Sigma-Aldrich Co., St. Louis, Missouri, USA) and cordycepin (C3394, Sigma-Aldrich Co., St. Louis, Missouri, USA), were used as standards for quality controls.

### STZ-induced diabetic mouse model

Thirty 8-week old male C57BL/6 mice (average weight = 20 ± 3 g) were purchased from the National Laboratory Animal Center (Taipei, Taiwan). The environment was maintained at 21 ± 3 °C, 50 ± 10 % relative humidity, and under a 12 h:12 h light/dark cycle with free access to food and water. All animal handling in this study are in accordance with a protocol approved by the Institutional Animal Care and Use Committee of National Taiwan University (IACUC approval NO. NTU-102-EL-37). All efforts were made to minimize animal suffering and to reduce the number of animals used, including that the animal sample size used for this current study was analyzed by G-power test with G*Power version 3.0.10.

The mice were first acclimatized for 7 days in the standard housing condition. After acclimatization, diabetes was induced by administrating streptozotocin (STZ) as previously described by Islam et al. [28, 29]. The STZ-induction mice were fasted for 6 h and before administered with 40 mg/kg STZ (S0130, Sigma-Aldrich Co., St. Louis, Missouri, USA) solution via intraperitoneal (IP). STZ was given once a day for 5 days with 2 subsequent days for recovery. A total of 30 mice were used in the present study with 24 mice as the STZ-induction group, while the remainder 6 served as the control group. A glucose tolerance test (GTT) was performed to confirm diabetes pathophysiology after STZ induction. STZ-induced mice were randomly divided into 3 groups (*n* = 8 mice/group) subjected to oral gavage with 100 μL solution of 25 mg/mL OFE, 2 mg/mL Rosi dissolved in PBS (R2408, Sigma-Aldrich Co., St. Louis, Missouri, USA) or PBS alone once per day for 28 days. An additional 6 mice without STZ induction were treated with PBS as the control group. Body weight and food intake were recorded daily during the experimental period. Mice are then sacrificed and islet cells were isolated and quantified as previously reported [30]. The subcutaneous white adipose tissue (SAT) was weighed and relative ratio of tissue-to-body weight was calculated. Moreover, blood samples were collected prior to sacrifice and stored at −80 °C for serological analyses, including mouse insulin ELISA kit (EMINS, Thermo Fisher Scientific, Waltham, Massachusetts, USA) and adiponectin ELISA kit (DY1119, R&D systems Ind., Minneapolis, Missouri, USA).

### Intraperitoneal glucose tolerance test (IPGTT)

**Fig. 1:**
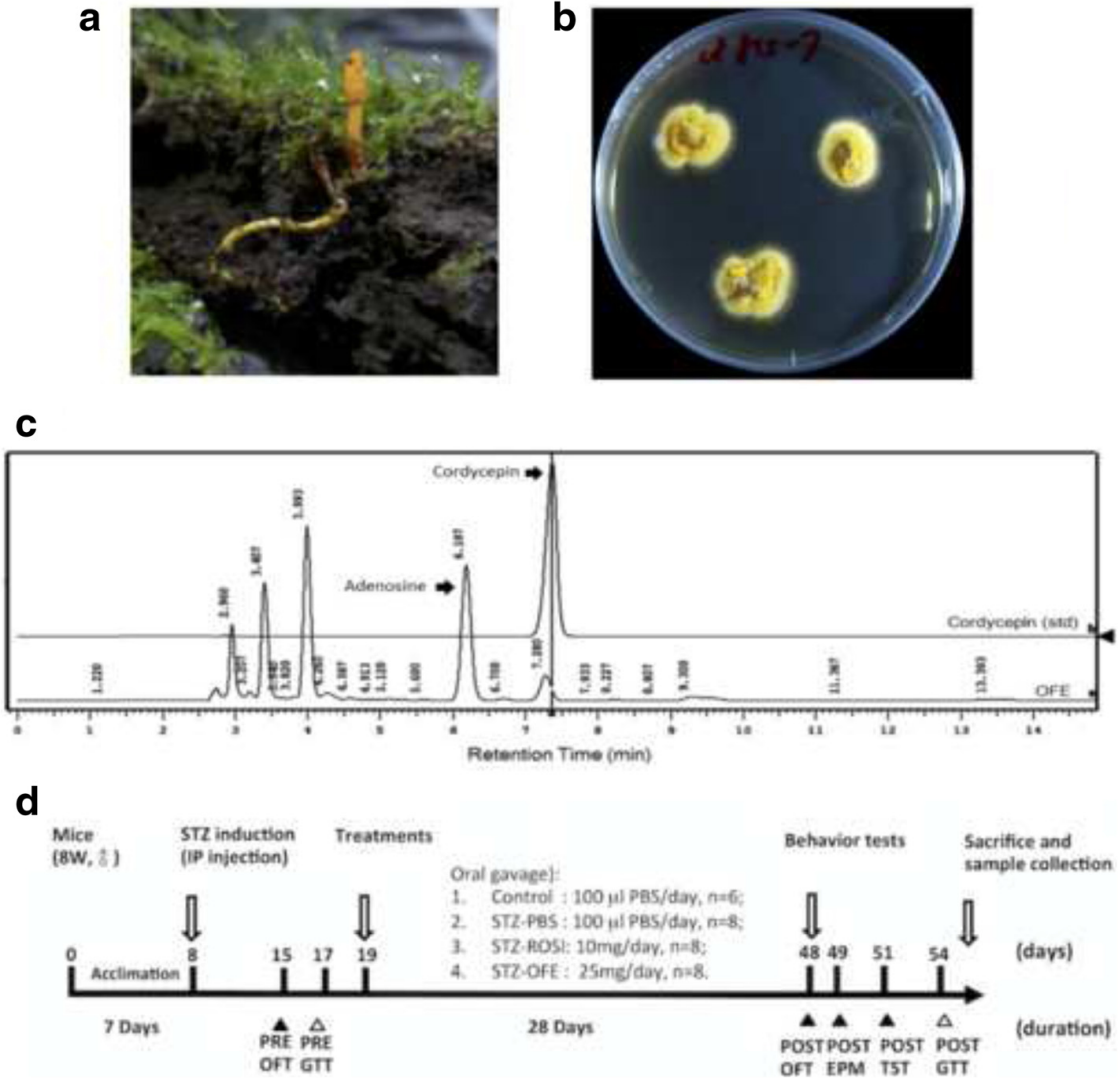
Experimental timeline and procedure*.* **a** *Ophiocordyceps formosana* (OF) was collected from fields in Taiwan. **b** Cultivation of OF mycelia on PDA plates with yellowish colonies. **c** Extracts of OF (OFE) were analyzed by a DAD-HPLC system. A representative HPLC profile of OFE allows quantitative analyses of the indicated components, including adenosine and cordycepin. **d** Scheme for experimental timeline and procedures. Eight week-old mice were first acclimated for 7 days prior to intraperitoneal (IP) injection with 40 mg/kg STZ solution for each mouse once a day for constitutive 5 days and recovery for 2 days. Six mice were injected with PBS as a sham-control group. To monitor anxiety and insulin resistance status for the STZ-treated and control group mice, at day 15, mice were conducted an anxiety behavior test by the open field test (OFT), and intraperitoneal glucose tolerance test (IPGTT) was performed before and after the above treatments (Day 17 and Day 54, respectively). The STZ-treated mice were then randomly divided into 3 different groups for oral gavage daily by 100 μL solution given of PBS, the 25 mg/mL OFE and 2 mg/mL Rosi, respectively. At day 48, several behavior tests, including OFT, EPM and TST, were used. All mice were then fasting for 6 h before evaluating insulin sensitivity by GTT. The mice were sacrifice after 1-day recovery from GTT

Glucose intolerance was measured by IPGTT as modified from Andrikopoulos et al. [31]. In brief, mice were fasted for 6 h prior to the 2 g/kg glucose administration by IP injection. IPGTT was performed before and after the above treatments (Fig. 1d, Day 17 and Day 54, respectively). Blood samples were collected from the tail at 0, 30, 60, and 120 min after glucose infusion to determine plasma glucose concentration utilizing a glucometer (Accu-Chek® active, Roche Diagnostics Ltd., Mannheim, Germany).

### Animal behavioral tests

A series of anxiety-like and depression-related behavior tests including open field test (OFT), elevated plus maze (EPM), and tail suspension tests (TST) are conducted to further characterize the depressive behavior associated with hyperglycemia as reported by previous literature [32–35].

An activity monitor enclosed in a sound-attenuated chamber equipped with ventilation was used to videotape all test sessions. Each mouse was individually placed in the center of an open-field apparatus (43.3 × 21.7 × 20.8 cm^3^, made of clear plexiglass) and allowed to explore freely for 60 min. Total travelled distance, duration, and velocity in the central and peripheral areas were analyzed by TopScan v2.0 software (Clever Sys Inc., Reston, VA, USA). The elevated plus maze is consisted of 2 open arms (30-L × 5-W cm^2^ in size) and 2 close arms enclosed by 50 cm high white acrylic walls. Open arms and close arms cross each other at right angle and is located 50 cm above the floor. All arms were made of white acrylic plates radiating from a central platform (5 × 5 cm^2^) to form a “+” sign. Each mouse was placed in the central platform area facing one of the closed arms. The motion trail and time spent in the apparatus were recorded for 5 min, and analyzed by TopScan v2.0 (Clever Sys Inc., Reston, VA, USA). Tail suspension test is used to assess the depressive behavior in animals. In brief, each mouse was taped by the tail (2–3 cm from the tip of the tail) and suspended for 6 min at 35 cm above the floor [36, 37]. The mouse was acoustically and visually isolated. The recorded data of all test sessions were used to calculate the immobility time, and subsequently the behavioral performance of each mouse was scored by three investigators. Immobility was defined as when all paws and the head of the mouse completely stop moving.

### Immunohistolochemical analyses

Subcutaneous white adipose tissues (SAT) were isolated and immediately fixed with 4 % paraformaldehyde, then embedded into paraffin. Immunohistochemical analyses were performed for examining adiponectin expressions as described by Schindler et al. [38]. Tissue sections were first deparaffinized, and subjected to antigen retrieval at 95 °C for 15 min in antigen retrieval buffer (ab93680; Abcam, Cambridge, Massachusetts). Endogenous peroxidase activity was blocked with 0.3 % H_2_O_2_ in 80 % methanol for 30 min. The sections were then blocked with 1 % BSA and incubated with an anti-mouse adiponectin antibody (ab22554; Abcam, Cambridge, Massachusetts) for 18 h at 4 °C. Subsequently, the sections were incubated with a goat anti-mouse secondary antibody (K500711, DakoCytomation Ltd., CA, USA) for 30 min and visualized using a DakoCytomation EnVision® + Duallink system HRP (DAB+) kit (K4065, DakoCytomation Ltd., CA, USA). Slides were counterstained with hematoxylin, rinsed in ddH_2_O for 10 min, and mounted with glycerin gelatin (GG1, Sigma-Aldrich Co., St. Louis, Missouri, USA). Representative 40X images were photographed and presented. Five areas of a section were used for the signal intensity quantification. The signal intensity of adiponectin expression in SAT was calculated using Image J, an image-analysis program (NIH, Bethesda, MD, USA) [39].

### Measurement of monoamine levels in mouse brains

The monoamine levels in various limbic regions were analyzed as described previously [40]. Briefly, brain was extracted and prefrontal cortex, striatum and hippocampus were isolated on an ice-chilled plate and weighed. The brain tissues were homogenized in 1 mL of extract solution consisting of 0.1 M HCl, 10^−7^ M ascorbic acid and 50 pg/μL isoproterenol. Homogenates were then centrifuged three times at 13200 rpm for 20 min at 4 °C and filtered through a 0.22 μm filter (Millipore, Bedford, USA). Supernatent was injected into a reverse C18 column (Hypersil GOLD aQ, 5 μm, 150 mm x 4.60 mm, Thermo Fisher Scientific, Inc., Massachusetts, USA). The concentrations of norepinephrine (NE, A7257, Sigma), dopamine (DA, H60255, Sigma), 3,4-dihydroxyphenylacetic acid (DOPAC, 850217, Sigma), 5-hydroxytryptamine (5-HT, H9523, Sigma), and 5-hydroxyindoleacetic acid (5-HIAA, H8876, Sigma) were measured by HPLC coupled with an electrochemical detector (P/N 70–9143 10 nA range, 1 Hz filter, and 0.7 V AppE cell ECD, Shiseido Co., Tokyo, Japan) equipped with an auto-sampler (WPS-3000, Shiseido Co., Tokyo, Japan). The mobile phase was 0.17 M NaH_2_PO_4_, 0.63 mM ethylenediaminetetraacetic acid (EDTA), 0.60 mM octane-1-sulfonic acid sodium salt, and 2 mM KCl in 20 % methanol, and adjusted to pH 3.30 with 85 % H_3_PO_4_. The flow rate was maintained in 0.6 mL/min. All the standards were purchased from Sigma (St. Louis, MO, USA) and dissolved in the extraction solution for HPLC. The standard curves ranged from 0.01 to 5.0 ppm, and the detection limit of this system was 0.1 pg for all samples.

### Statistical analyses

Statistical significance was tested by one-way ANOVA and Tukey’s multiple comparison test (SAS institute, Cary, NC, USA). Data were expressed as mean ± S.E.M. *p* values ≤ 0.05 were considered statistically significant. The body weights were compared by the Student’s *t* test. Data were expressed as mean ± S.E.M. # represents *p* ≤ 0.05, and *** represents *p* ≤ 0.001.

## Results

### Extraction and chemical analyses of Ophiocordyceps formosana

*Ophiocordyceps formosana* (*Cordyceps s.l.*) was established, cultivated (Fig. 1a and b) then water extracted for bioactive compound analyses by HPLC (Fig. 1a-c). Like other known medicinal *Cordyceps spp.*, the extract of *O. formosana* contained adenosine and cordycepin (Fig. 1c), potent bioactive components for medicinal use. Hence, we sought for its potential benefit on diabetic complications.

### OFE ameliorates the characteristics of STZ-induced diabetic mice

**Fig. 2:**
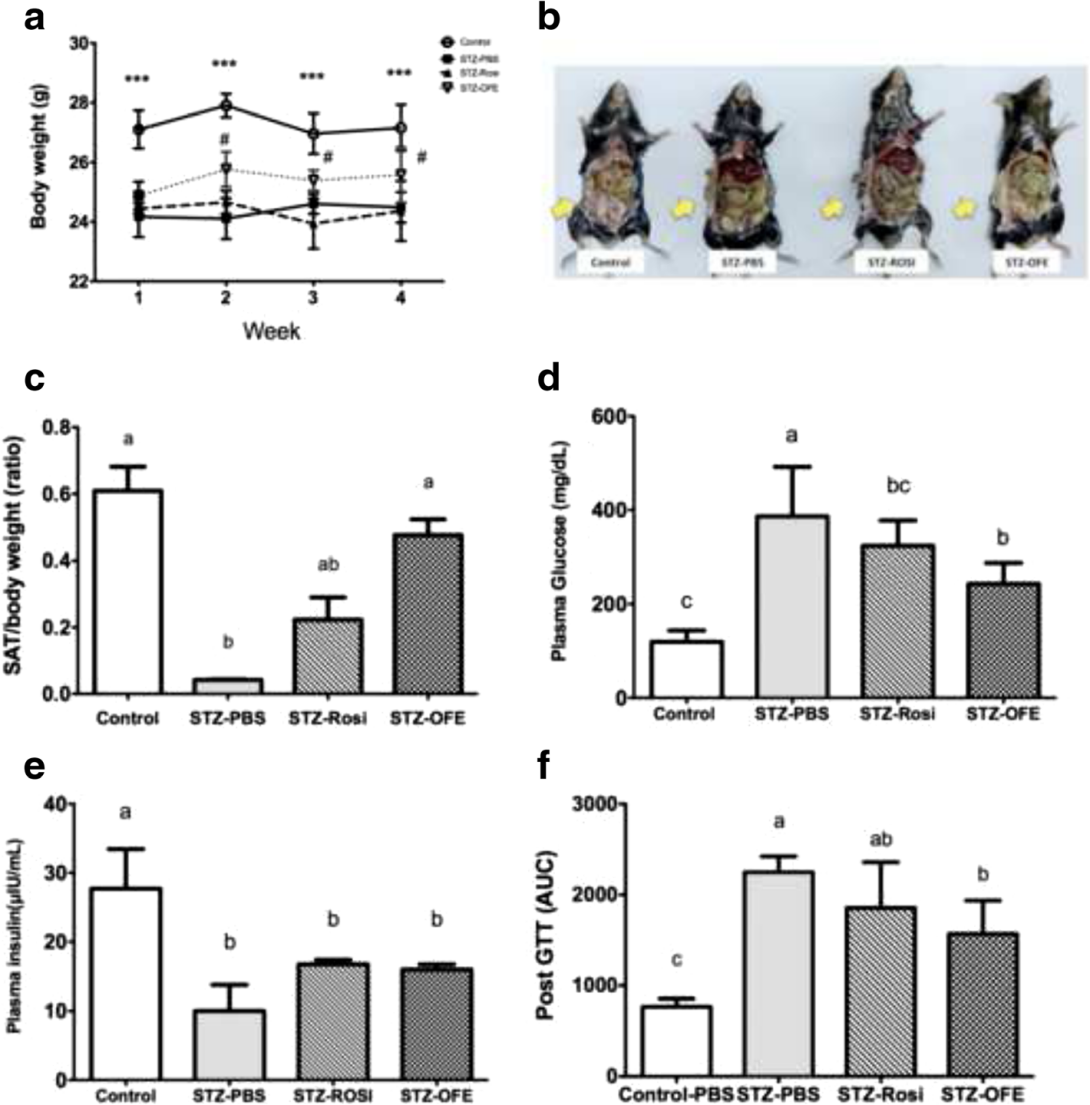
*Ophiocordyceps formosana* ameliorated the hyperglycermic characteristic of STZ-induced diabetic mice. **a** Changes in body weight of mice. **b** Morphological appearance of visceral adiposity in mice. The arrows show the visualized adipose tissues. **c** The subcutaneous fat (SAT) to body weight ratio. **d** The glucose concentration in plasmas at the end of the experiment. **e** The insulin concentration in plasma at the end of the experiment. **f** The area under curve (AUC) of glucose tolerance test (GTT) after treatments. Data were expressed as mean ± S.E.M. # represents *p* ≤ 0.05, STZ-PBS vs. STZ-OFE and *** represents *p* ≤ 0.001, control vs. STZ-PBS, STZ-Rosi and STZ- OFE. Results of two groups were compared by the Student’s *t* test. One way ANOVA followed by Tukey’s post hoc test was performed for multiple comparisons. Mean in a row with different superscripts is significantly different (*p* ≤ 0.05). Values are mean ± SEM, *n* = 6 in control group and *n* = 8 in the all STZ-induced groups

To examine the medicinal benefit of the water extract of *O. formosana* on diabetes, an STZ-induced diabetic animal model was employed. Consistent with features of type I diabetes, STZ-induced diabetic mice displayed body weight loss (Fig. 2a) and a fasting plasma glucose value over 200 mg/dL (control = 119.5 ± 24.2 mg/dl and STZ-PBS = 392.2 ± 38.7 mg/dl, *p* ≤ 0.001) (Fig. 2d). In addition, the pancreatic β cell number was diminished in STZ-induced diabetic mice, but neither Rosi nor OFE treatment could rescue the number of β cells (data not shown). However, the body weight was regained and the subcutaneous adipose tissues (SAT) accumulated in the OFE-treated group compared to the PBS treated group. The OFE treated mice exhibited a better outcome than the Rosi-treated mice (Fig. 2b and c). Furthermore, the plasma glucose and area under curve (AUC) of the plasma glucose tolerance test (GTT) in the OFE treated STZ-induced diabetic mice was reduced, indicating that OFE could partially alleviate hyperglycemia induced by STZ (Fig. 2d). However, the concentration of plasma insulin did not show signs of recovery in OFE groups (Fig. 2e). These results suggest that OFE is potentially capable of ameliorating pathophysiological complications of STZ-induced diabetic mice, but not mediate insulin recovery.

### OFE increased adiponectin expression in plasma and adipose tissue

**Fig. 3:**
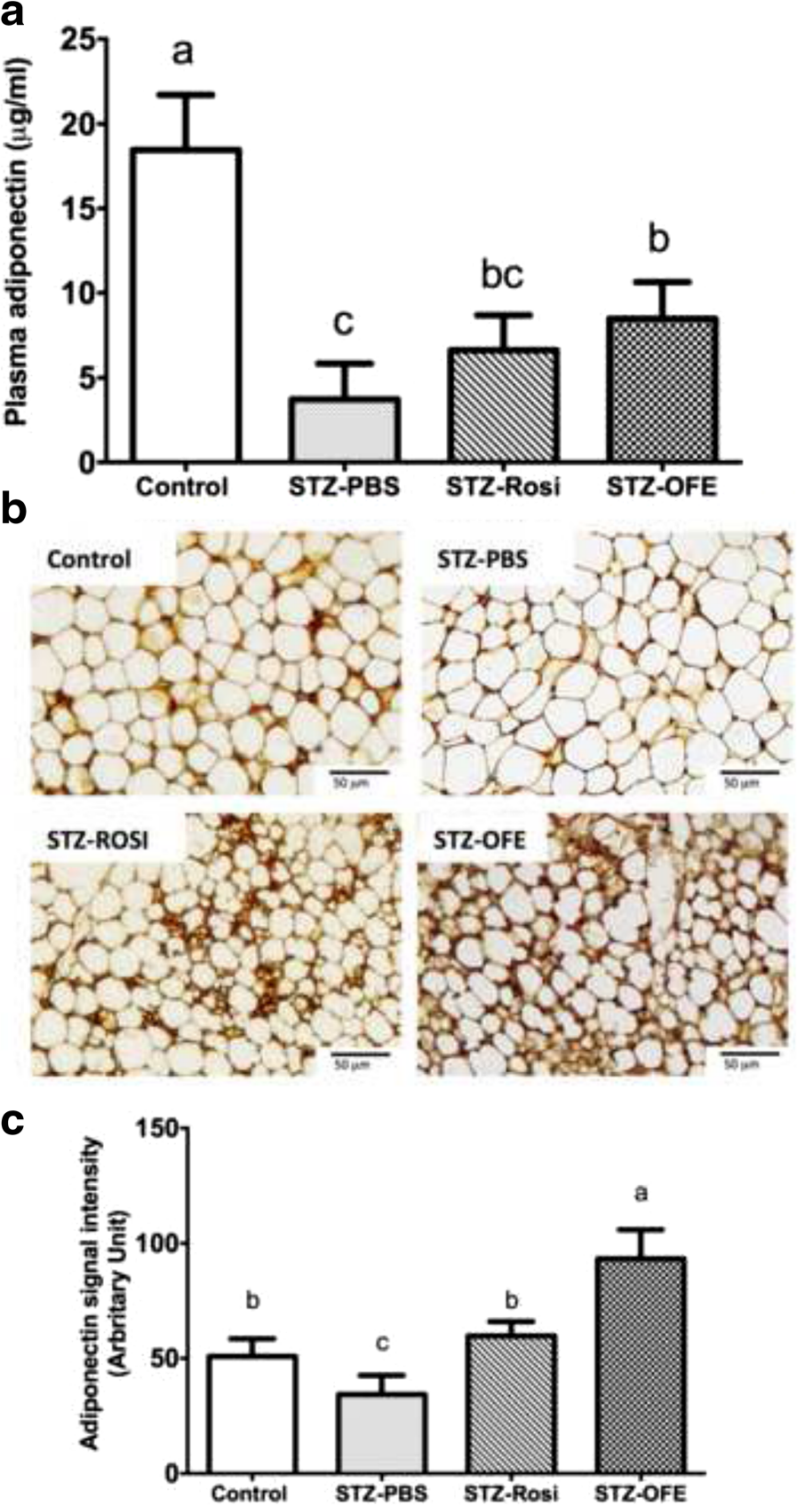
*Ophiocordyceps formosana* upregulated adiponectin expressions in plasma and adipose tissues. **a** The levels of plasma adiponectin in different STZ-induced treated groups. **b** Immunohistochemical staining for adiponectin in adipose tissues. **c** The adiponectin-positive area of each treated group was quantified by Image J. Scale bar = 50 mm, under a 400x microscope. One-way ANOVA followed by Tukey’s post hoc test was performed for multiple comparisons. Mean in a row with different superscripts is significantly different (*p* ≤ 0.05). Values are mean ± SEM, *n* = 6 in control group and *n* = 8 in all STZ-treated groups

Adiponectin is an adipokine highly correlated with fat biogenesis and insulin sensitivity [4]. The level of adiponectin in plasma was decreased by STZ compared with the normal control group (Fig. 3a). As expected, the level of plasma adiponectin was significantly elevated by OFE treatment compared to PBS treatment. However, OFE or Rosi treatment failed to rescue plasma adiponectin level in comparison to the non-diabetic control group (Fig. 3A). The enlarged adipocyte size in STZ-diabetic mice was reduced in Rosi-treated mice and to a greater extent in OFE-treated mice (Fig. 3b). Moreover, STZ treatment lowered adiponectin level in subcutaneous adipose tissue, while Rosi treatment elevated adiponectin level and OFE treatment showed a greater increase compared to the non-diabetic controls (Fig. 3c). Taken together, our data imply that OFE may up-regulate the expression of adiponectin in SAT and plasma leading to the promotion of biogenesis of SAT in the diabetic mice.

### OFE reduces the anxiety and depression-like behaviors in STZ-induced diabetic mice

**Fig. 4:**
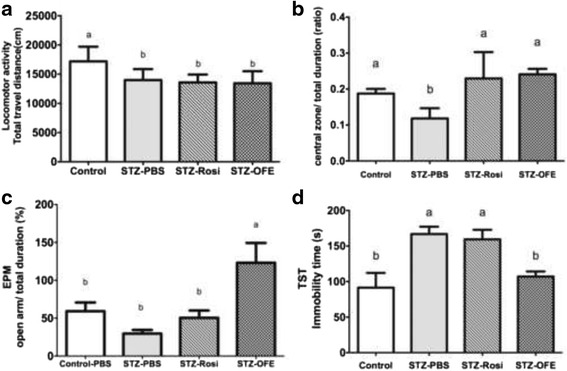
*Ophiocordyceps formosana* improved depressive-like behaviors in STZ-induced diabetic mice. **a** Locomotor activity in the open filed test (OFT) (**b**) The ratio of retention time in the central zone to total duration time in the OFT. **c** The ratio of the retention time in the open arm to total duration of the equipment (%) in the elevated plus maze (EPM). **d** The immobility time in the tail suspension test (TST). * represent *p* ≤ 0.05, Control-PBS vs. STZ-PBS. One way ANOVA followed by Tukey’s post hoc test was performed for multiple comparisons. Mean in a row with different superscripts is significantly different (*p* ≤ 0.05). Values are mean ± SEM, *n* = 6 in control group and *n* = 8 in all STZ-treated groups

To evaluate whether OFE confers benefits to the aforementioned psychological complications, we conducted animal behavioral tests, including open filed test (OFT), elevated plus maze (EPM) and tail suspension test (TST) [35]. The STZ-induced diabetic mice displayed anxiety- and depression-related behaviors compared to control group (Fig. 4), and the total travel distance decreased by STZ induction (Fig. 4a). Conversely, the STZ-induced diabetic mice treated with either Rosi or OFE showed an increased duration time spent in the central zone of the OFT (Fig. 4b). Moreover, the retention time spent in the open arms of the EPM was extended in the STZ-OFE group, indicating that OFE promoted exploratory behaviors (Fig. 4b and c). Similarly, results from the tail suspension test showed an extended motionless duration time in the STZ-PBS group (Fig. 4d), suggesting that the STZ-induced diabetic mice expressed depression-like behaviors. In contrast, the immobility time of the OFE group was similar to that of the non-diabetic mice (Fig. 4d). Collectively, our data demonstrated that OFE is potent to ameliorate anxiety and depression-related psychological complications associated with type I diabetes.

### Evaluation of various neurotransmitters levels in different brain regions of STZ-induced diabetic *mice treated by OFE*

To elucidate the underlying mechanisms of OFE on alleviating the aforementioned psychological complications, levels of neurotransmitters (i.e., DA and 5HT) and their metabolites (i.e., DOPAC and 5HIAA) in the brain, including the hippocampus, the striatum and the prefrontal cortex, were measured by ECD-HPLC (Table 1). STZ-induced diabetic mice demonstrated reduced levels of DA, DOPAC, 5HT and 5HIAA in the three brain regions compared to control mice (Table 1). Intriguingly, neurotransmitter levels were recovered by either Rosi or OFE treatment, especially in the frontal cortex and striatum (Table 1), which may contribute to the changes in behaviors observed after Rosi and OFE treatment (Fig. 4). Noticeably, the amount of norepinephrine (NE) in the frontal cortex and hippocampus were increased by STZ-treatment, but not in the striatum. Our data suggest that OFE can alter the expression levels of neurotransmitters in dopaminergic and serotonergic pathways.

**Table 1:** Expressions of various neurotransmitters, including the frontal cortex, striatum and hippocampus of brain, in mice

Brain Cortex	Treatment	Neurotransmitter (ng/g brain tissue)				
		DA	DOPAC	5HT	5HIAA	Norepinephrine
Frontal Cortex	Control	1277.7 ± 332.8^b^	741.7 ± 115.5^b^	797.3 ± 72.8^a^	863.0 ± 47.2^a^	409.1 ± 107.3^c^
	STZ-PBS	238.4 ± 27.00.2^b^	175.9 ± 55.5^c^	323.3 ± 93.4^c^	99.7 ± 11.5^c^	685.6.9 ± 34.4^b^
	STZ-Rosi	851.5 ± 307.3^b^	615.3 ± 54.2^b^	594.5 ± 220.3^bc^	283.7 ± 39.9^b^	939.2 ± 81.8^a^
	STZ-OFE	2343.2 ± 824.4^a^	1467.4 ± 136.6^a^	931.1 ± 45.6^a^	939.5.7 ± 59.8^a^	861.33 ± 84.4^a^
Striatum	Control	986.8 ± 73.2^b^	1013.1 ± 101.6^ab^	936.7 ± 93.8^a^	863.0.0 ± 47.2^b^	916.52 ± 49.9^a^
	STZ-PBS	806.1 ± 138.3 ^b^	681.6 ± 61.2^c^	583.1 ± 97.4^b^	481.0 ± 33.9^c^	613.7 ± 26.2^b^
	STZ-Rosi	1185.3 ± 423.9^b^	797.6 ± 103.1^bc^	613.8 ± 129.7^b^	491.7 ± 20.2^c^	731.8 ± 55.9^b^
	STZ-OFE	2760.7 ± 323.0^a^	1069.9 ± 120.8^a^	693.2 ± 96.7^ab^	716.7 ± 57.1^a^	679.6 ± 65.7^b^
Hippocampus	Control	288.5 ± 72.8^a^	111.7 ± 30.5^b^	1041.9 ± 103.6^b^	1040.7 ± 71.6^a^	257.6 ± 63.9^c^
	STZ-PBS	84.6 ± 5.1^bc^	129.0 ± 46.9^b^	620.2 ± 66.2^c^	219.0 ± 5.9^b^	423.5 ± 85.8^bc^
	STZ-Rosi	19.9 ± 6.2^c^	49.1 ± 28.0^bc^	864.3 ± 41.0^b^	185.4 ± 63.2^b^	702.9 ± 12.1^a^
	STZ-OFE	255.1 ± 56.3^a^	317.4 ± 121.3^a^	1622.7 ± 83.0^a^	280.8 ± 61.5^b^	592.2 ± 107.9^ab^

DA: dopamine; DOPAC: 3,4-dihydroxy phenlactic acid; 5-HT: 5-hydroxytryptamine; 5-H1AA: 5-hydroxy indole acetic acid. One way ANOVA followed by Tukey’s post hoc test was performed for multiple comparisons. Means in a row with different superscripts, such as ^a^, ^b^, ^c^, ^ab^, ^bc^, is significantly different (*p* <= 0.05). Values are present as mean ± SEM, *n* = 6 in control group and *n* = 8 in the ogher groups

## Discussion

**Fig. 5:**
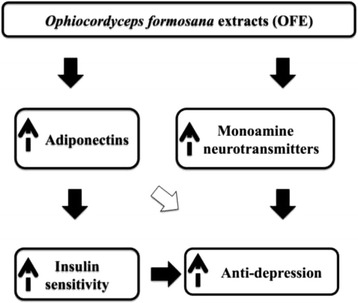
Putative mechanisms for OFE in improving insulin resistance and depression. OFE promotes the expression of adiponectin, which is an adipokine involved in insulin sensitivity and anti-depression. In addition, the neurotransmitters (5-HT and dopamine) in brain regions were also elevated by OFE, which also leads to anti-depression

Type 1 diabetic (T1D) patients suffer weight loss, increase fluid consumption and urination, as well as several mental complications, such as irritability, anxiety, and depression [41]. Unfortunately, current therapeutics like, β*-*cell transplantation, immunosuppressive regimens for pancreas transplantation or daily insulin injection [1], are served to alleviate most of the complications induced by T1D, but such treatments are limited and inefficient,. Hence, our current study employed a diabetic animal model induced by STZ to mimic the symptoms of hyperglycemia and depression as observed in T1D patients [42]. We found that the OFE treatment elevated adiponectin levels, various brain neurotransmitter expressions, and ameliorated various diabetes-related complications, including hyperglycermia and anxiety/depression (Fig. 5). Our results indicate the pharmacological potential for a newly established medicinal fungus, *Ophiocordyceps formosana*, as an alternative and complementary medicine.

Presently, several oral therapeutic regimens for treating diabetes-related hyperglycemia and depression have been widely developed and used. For instance, thiazolidinedione (TZDs) drugs, such as rosiglitazone (Rosi), have been reported to effectively control hyperglycemia in T2D [43]. In addition, Rosi has been shown to ameliorate depressive behaviors by reduction of plasma corticosterone levels [44]. However, several undesirable side effects, such as stomach pain, diarrhea, heart failure and edema, are often observed [45, 46]. Likewise, T1D patients show symptoms of depression can be treated with oral antidepressants [47, 48]. For instance, selective serotonin reuptake inhibitors (SSRIs) are the most widely prescribed antidepressants in spite of numerous adverse side effects [12]. Thus, there remains a need for regimens that can ameliorate the symptoms of T1D with fewer side effects. A natural product derived from an *Ophiocordyceps sinensis* relative, *O. formosana* has relatively lower toxicity in vitro [26] and as shown in this current study seems to be effective in the STZ mouse model for T1D. However, more studies remain needed to evaluate its safety and indications.

Like *Ophiocordyceps sinensis* [49], *O. formosana* exhibited a beneficial improvement in body weight of STZ-induced mice and an increase in SAT (Fig. 2). The extended plasma GTT in STZ-induced diabetic mice was partially corrected by either Rosi or *O. formosana* treatment (Fig. 2d). These results suggest that *O. formosana* not only promotes glucose metabolism, but also increases energy utilization in mice. Rosi is a PPAR-γ agonist capable of increasing adiponectin expression, synthesis and release [50]. Although adiponectin is exclusively secreted from adipocytes, it is a circulating hormone whose level in plasma is positively correlated with insulin sensitivity and inversely associated with obesity and T2D [21, 51]. Despite being highly expressed in most fat tissues, the level of adiponectin is lower in visceral adipose tissues than in subcutaneous abdominal adipose tissues (SAT), suggesting that SAT may be the major contributor to circulating adiponectins [52]. Consistently, our results showed that mice treated with 25 g/ml/day of OFE for 28 days had increased SAT as well as adiponectin in plasma and SAT (Fig. 3). We hypothesized that *O. formosana* ameliorates insulin resistance via up-regulation of adiponectin.

In addition to improvements in glucose sensitivity and adipogenesis, an increase in plasma adiponectins is reportedly associated with reduced depressive disorders in patients with diabetes [53, 54]. The intracerebroventricular infusion of recombinant globular adiponectin or recombinant full-length adiponectin reduced depressive-like behavioral effects in normal weighted mice [21]. Furthermore, adiponectins alleviate depression-like behaviors attributed to their neuroprotective activity and hippocampal neurogenesis modulation in mice [55]. Our observations demonstrated that the increase in adiponectin in plasma and adipose tissues by *O.formosana* may have attributed to the improvement in behavioral patterns in our mice treated with OFE.

*O. formosana* modulated monoamine neurotransmitters, including dopamine, 5-HT and norepinephrine (NE) in various brain regions, including the hippocampus, amygdala and striatum. These brain regions take part in regulating motion and rewarding as well as executive functions; therefore, their dysfunction has been implicated in depression [56, 57]. Depressive illness is linked with an insufficiency of brain monoamine neurotransmitters including dopamine, 5-HT and norepinephrine (NE) in these brain regions [12, 58]. Tail suspension test utilized to assess antidepressant-like activity in mice by observing their behavior under the inescapable stress of being suspended above ground [59]. Administration of OFE decreased immobility time and was accompanied by increased amounts of DA and 5-HT in the hippocampus and frontal cortex (Fig. 4d and Table 1). The striatum is essential in mediating anxiety and reducing motivation in patients with depression [60]. Two anxiety-related tests, EPM and OFT, displayed increases in retention time in the open arms and the central zone, respectively, in OFE-treated STZ-induced mice along with increased DA and 5-HT in the striatum (Table 1). Although there are conflicting reports, we found that the level of NE was increased in STZ-induced diabetic mice and is consistent with previous reports [61, 62]. The locomotor activity showed no significance between groups (data now shown), suggesting that the improvement of anti-depressive-like behaviors by OFE was not caused by hyperactivity. Taken together, *O. formosana* ameliorates both anxiety-like and depressive-like behaviors in STZ-induced diabetic mice. The increased levels of various neurotransmitters in some brain regions in these mice suggest that the changes in behaviors may result from modulation of neurotransmitters.

Rosiglitazone (Rosi) has been applied for the clinical treatment of T1D patients with depression [63]. After 12 weeks of treatment with Rosi, severity of depression based upon the Hamilton Depression Rating Scale and the Clinical Global Impression Scale was improved [15]. The results indicated that improvement of insulin resistance is a prospective treatment for alleviation of depression. Recently, an emerging relationship between inflammation and depression has led to the use of anti-inflammatory drugs to ameliorate the symptoms of depression [64, 65]. Here, we provide a new approach to ameliorate insulin insufficiency and depression mediated by *Ophiocordyceps formosana*; the mechanism was suggested to be via the upregulation of adiponectins. Although some minor components of *Cordyceps spp.*, such as cordycepin, polysaccharides and trace elements like vanadium, are reported to be responsible for the antidepressant-like activities [25], further investigation is necessary to identify the constituents of *Ophiocordyceps formosana* rendering the anti-hyperglycemic and anti-depression activities.

## Conclusion

To our knowledge, our current study demonstrated, for the first time, the medicinal merit of *Ophiocordyceps formosana* on T1D and hyperglycemia-induced depression. Our results revealed OFE improved glucose utilization by promoting adiponectin expression in STZ-induced diabetic mice. The administration of OFE also modulated the expression of monoamine neurotransmitters and their metabolites (i.e., 5-HT and DOPA). Collectively, this study provided a potential usage of *O. formosana* extracts to counteract hyperglycemia and depression complications.

## Abbreviations

5-HT: 5-hydroxytrptamine
DOPA: Dopamine
EPM: Elevated plus maze
IPGTT: Intraperitoneal glucose tolerance test
NE: Norepinephrine
OFE: Ophiocordyceps Formosana extract
OFT: Open filed test
Rosi: Rosiglitazone
SAT: Subcutaneous white adipose tissue
SSRI: Serotonin re-uptake inhibitor
STZ: Streptozoticin
T1D: Type 1 diabetes
T2D: Type 2 diabetes
TCA: Tricyclic antidepressants
TCM: Traditional Chinese medicine
TST: Tail suspension test
TZD: Thiazolidinedione
